# Achtsamkeit in der Schule (AISCHU) – Evaluation der Weiterbildung für Lehrkräfte zur Stressreduktion

**DOI:** 10.1007/s11553-021-00870-9

**Published:** 2021-07-05

**Authors:** Jana Kraft, Vera Kaltwasser, Niko Kohls

**Affiliations:** 1 Nürnberg, Deutschland; 2 Frankfurt, Deutschland; 3 Hochschule Coburg, Friedrich-Streib-Str. 2, 96450 Coburg, Deutschland

**Keywords:** Selbstregulation, Gesunheitsförderung, Schule, LehrerInnen, Burn-out, Self-regulation, Health promotion, Schools, Teachers, Psychological burnout

## Abstract

**Hintergrund:**

Positive Effekte von achtsamkeitsbasierten Programmen im Kontext Schule wurden bereits vielfältig belegt. Das in dieser Studie evaluierte Konzept Achtsamkeit in der Schule (AISCHU) richtet sich an Lehrkräfte und SchülerInnen und soll deren Stresswahrnehmung und Selbstregulationsfähigkeit schulen. Im Fokus dieser explorativen Studie steht erstmals der präventive Nutzen der AISCHU-Weiterbildung für Lehrkräfte und deren psychische Gesundheit.

**Methodik:**

Etablierte Messinstrumente bezüglich Stresserleben (PSQ), Burn-out-Gefährdung („Tedium measure“ (TM)), Wohlbefinden (WHO-5) und Achtsamkeit (FFA, MAAS) wurden im Prä-Post-Vergleich eingesetzt. Die Daten beziehen sich auf 36 Lehrkräfte unterschiedlicher Schulformen.

**Ergebnisse:**

Es ergaben sich hinsichtlich aller untersuchten Messgrößen signifikante Verbesserungen mit z. T. großen Effektstärken.

**Schlussfolgerung:**

In dieser explorativen Pilotstudie zu AISCHU für Lehrkräfte konnten erstmals vielversprechende Hinweise auf die Wirksamkeit im Sinne von Stressreduzierung, Burn-out-Risikominimierung sowie Verbesserung der Lebensqualität bei belasteten LehrerInnen beobachtet werden.

**Zusatzmaterial online:**

Zusätzliche Informationen sind in der Online-Version dieses Artikels (10.1007/s11553-021-00870-9) enthalten.

## Abstract (en)

**Background:**

The positive effects of mindfulness-based interventions in the school context have already been empirically demonstrated. The “Mindfulness in Schools” concept (AISCHU) evaluated in this study was designed for both teachers and students to improve their stress perception and self-regulation skills. This exploratory study is the first to focus on the preventive benefits of professional development (AISCHU) for teachers and their mental health.

**Methods:**

Established measurement instruments regarding stress experience (PSQ), burnout risk (TM), well-being (WHO-5), and mindfulness (FFA, MAAS) were used in the pre–post comparison. In all, 36 teachers working at different types of schools participated in the program.

**Results:**

There were significant improvements with large effect sizes with regard to all parameters examined.

**Conclusions:**

In this explorative pilot study of AISCHU for teachers, promising indications of effectiveness in terms of stress reduction, burnout risk minimization, and improvement of quality of life among stressed teachers could be observed for the first time.

Wegen der hohen Belastung von SchülerInnen und LehrerInnen im Setting Schule wird zunehmend die Bedeutung von Selbstregulationskompetenzen für die Förderung von Gesundheit, Wohlbefinden, Lernerfolg bzw. Arbeitszufriedenheit thematisiert. Weil damit zusammenhängende Erkenntnisse bislang nicht systematisch in Aus- bzw. Weiterbildungskonzepte umgesetzt wurden, wurde das Programm „Achtsamkeit in der Schule“ (AISCHU) konzipiert. Es dient der Achtsamkeitsschulung für Lehrkräfte und SchülerInnen. Diese Studie untersucht erstmals den gesundheitsförderlichen Nutzen der Intervention für die psychische Gesundheit von LehrerInnen.

## Hintergrund

Stress im lebensweltlichen Setting Schule wird nicht nur bei SchülerInnen sondern zunehmend auch bei Lehrkräften thematisiert. In der Folge der Coronakrise hat die Belastung noch einmal zugenommen. Eine Ende 2020 veröffentlichte Befragung an über 2300 Lehrkräfte in NRW zeigte auf, dass 60 % der Befragten das Unterrichten im Vergleich zum Vorjahr als deutlich anstrengender beurteilten und 28 % berichteten über ausgeprägte emotionale Erschöpfung [6]. Vor allem zur Vermittlung von Stressbewältigungskompetenzen, aber auch zur Verbesserung der Beziehungsqualität finden achtsamkeitsbasierte Programme zunehmend Einzug in den Schulkontext [4, 20]. Verschiedene Konzepte richten sich hier sowohl an Lehrkräfte wie auch an SchülerInnen. Durch Metaanalysen ließen sich bereits in mehrfacher Hinsicht günstige Effekte belegen. Für Lehrkräfte zeigen sich positive Auswirkungen in Bezug auf Stress- und Emotionsregulation. Achtsamkeitsbasierte Programme können Selbstwirksamkeit, Resilienz, Achtsamkeit und Selbstmitgefühl steigern [11] und das Burn-out-Risiko reduzieren [13]. Darüber hinaus können achtsamkeitsbasierte Programme auch bei SchülerInnen Aufmerksamkeitsprozesse, kognitive Kontroll- und Leistungsfähigkeit fördern und störendem Verhalten entgegenwirken [3, 20].

Bereits vor einigen Jahren entwickelte eine der Autorinnen (VK) das Trainingskonzept „Achtsamkeit in der Schule“ (AISCHU). Mit AISCHU werden Inhalte des klassischen MBSR-Konzepts („mindfulness-based stress reduction“) nach Jon Kabat-Zinn in adaptierter Form in den Schulunterricht integriert, um SchülerInnen altersgerecht in Achtsamkeit zu schulen [10]. Nachdem die Wirksamkeit für SchülerInnen in 2 Studien überprüft wurde [10, 19], wurde ebenfalls ein Konzept „Achtsame Acht Wochen“ speziell für Lehrkräfte entwickelt [8, 9]. Mit diesem Programm sollen durch Entwicklung einer eigenen Achtsamkeitspraxis die Selbstregulationsfähigkeit der Lehrkräfte und somit auch deren Stressbewältigungsfähigkeiten gefördert werden. Beide Programme wurden schließlich zu einem schlüssigen Gesamtkonzept weiterentwickelt: Zunächst sollen die LehrerInnen selbst ihre Achtsamkeit und damit auch Stressbewältigungs- und Beziehungsfähigkeit schulen, um die gelernten Inhalte und Methoden dann auch an ihre SchülerInnen zu vermitteln. Diese von Zenner [20] vorgeschlagene Vorgehensweise wurde in ähnlichen Konzepten bisher nicht umgesetzt [2, 7]. Die so entstandene einjährige Weiterbildungskomponente speziell für Lehrkräfte – ebenfalls genannt AISCHU – wird erstmals in einer explorativen Pilotstudie evaluiert.

## Fragestellung

Nachdem bereits positive Effekte der AISCHU-Intervention auf die Stresswahrnehmung und -bewältigung bei SchülerInnen empirisch gesichert werden konnten [10, 19], soll nun auch die gesundheitsbezogene Wirksamkeit der entsprechenden Weiterbildungskomponente für Lehrkräfte in einer explorativen Pilotstudie untersucht werden. Die zugrunde liegende Hypothese der Studie ist, dass sich – im Einklang mit bisherigen Studien – das subjektive Stressempfinden, die individuelle Burn-out-Gefährdung, das allgemeine Wohlbefinden sowie die Achtsamkeit der teilnehmenden Lehrkräfte im Prä-Post-Vergleich unter Berücksichtigung von Geschlechtsaspekten als Folge verbesserter Selbstregulationsfähigkeit signifikant verbessern.

## Methodik

### Inhalte und Ablauf der Weiterbildungsreihe

Die Lehrerweiterbildung AISCHU orientiert sich an einem klassischen achtsamkeitsbasierten Stressmanagementprogramm, das sowohl aus psychoedukativen wie auch erfahrungsbasierten Komponenten besteht. Dabei werden nicht nur neurowissenschaftliche Erkenntnisse der Stress- und Aufmerksamkeitsforschung vermittelt, sondern diese auch über geeignete, alltagskompatible Körper- und Bewusstseinsübungen trainiert. Der Schwerpunkt der Weiterbildung liegt auf der Verbesserung der Selbstregulationsfähigkeit, weswegen Selbstreflexion, Stressbewältigungsstrategien, Resilienz, Burn-out-Prophylaxe, Selbstfürsorge und Beziehungsfähigkeit vermittelt werden. Genauere Inhalte und Ablauf sind unter www.aischu.de nachzulesen.

Die AISCHU-Weiterbildung für Lehrerkräfte wurde mit den LeiterInnen der regionalen Lehrerfortbildung des staatlichen Schulamts Frankfurt am Main in Zusammenarbeit mit der Referentin (VK) auf der Basis bestehender Konzepte [8, 9] entwickelt und in Frankfurt und Berlin im Zeitraum von März 2019 bis Januar 2020 durchgeführt. In Frankfurt wurde das Programm in Kooperation mit dem dortigen Schulamt und dem Institut für Achtsamkeit, Verbundenheit und Engagement (AVE) durchgeführt. In Berlin handelte es sich um eine kostenpflichtige Fortbildung des gemeinnützigen Vereins „Achtsamkeit für Kinder und Jugendliche“ (AKiJu), der ein Tochterverband des MBSR-Verbands ist. Die Einladung zur Fortbildung erfolgte entsprechend durch das Staatliche Schulamt Frankfurt am Main an 149 SchulleiterInnen der Stadt. Eine Anmeldung für die Berliner Fortbildung über die Homepage des AKiJu war hingegen für LehrerInnen aus allen Bundesländern möglich.

Die insgesamt 60 Weiterbildungsstunden wurden in Frankfurt aus organisatorischen Gründen im Rahmen eines Einstiegswochenendes sowie drei Ganztages- und fünf Halbtagesterminen vermittelt. In Berlin wurde dies an vier Wochenendterminen zu je zwei ganzen und einem halben Tag durchgeführt. Zwischen den Terminen sah das Konzept einen wöchentlichen Erfahrungsaustausch zwischen den TeilnehmerInnen vor.

### Rekrutierung der TeilnehmerInnen

Die Teilnahme an der wissenschaftlichen Evaluation der Weiterbildung war für die Lehrkräfte freiwillig. Die Teilnehmenden wurden darauf hingewiesen, dass die Evaluation ein wichtiger Bestandteil zur Qualitätssicherung der Maßnahme ist. Sie wurden darüber informiert, dass alle Daten – aufgrund der Notwendigkeit Zeitreihendaten zuordnen zu können in pseudonymisierter Form – auf freiwilliger Basis gesammelt und analysiert werden. Dadurch konnten alle angemeldeten Lehrkräfte auch als TeilnehmerInnen für die Studie gewonnen werden. Alle Lehrkräfte haben an mindestens acht von zehn, die Mehrheit (52 %) sogar an allen Terminen teilgenommen.

### Datenerhebung und Messinstrumente

Die Seminare fanden im Zeitraum von März 2019 bis Januar 2020 statt und wurden im Präsenzformat durchgeführt. Dabei wurden die TeilnehmerInnen jeweils vor dem ersten (t0) sowie nach dem letzten (t1) der zehn Termine schriftlich am Veranstaltungsort befragt. Die Fragenbatterie beinhaltete folgende psychometrisch validierte Instrumente:

- „*Perceived stress questionnaire*“* (PSQ, *[5]) misst das subjektive Stressempfinden mit den Subfaktoren Sorgen, Anspannung, Freude und Anforderungen auf einer Skala von 1,00 („nie“) bis 4,00 („fast immer“).
- „*Tedium measure*“* (TM*, [16]) erfasst die Burn-out-Gefährdung anhand der Häufigkeit von Symptomen körperlicher, emotionaler und mentaler Erschöpfung auf einer Skala von 1,00–7,00. In der Validierungsstudie wurde ein Mittelwert von 2,96 (SD = 0,75) festgestellt [17]. Werte ab 3,00 können als kritische Stressbelastung, Werte ab 5,00 als deutliche Burn-out-Zeichen interpretiert werden.
- *WHO‑5 Wohlbefindensindex *[1]: Dieser Kurzfragebogen dient der Erfassung des Wohlbefindens bzw. der Lebensqualität. Die möglichen Summenwerte reichen von 0–25, wobei ein Indexwert < 13 ein reduziertes Wohlbefinden anzeigt.
- „*Mindful attention and awareness scale*“* (MAAS, *[15]) misst das Ausmaß der Unachtsamkeit im Alltag und wird hier invertiert als niedrige (1,00) bis hohe (4,00) Achtsamkeit dargestellt.
- *Freiburger Fragebogen zur Achtsamkeit (FFA, *[12, 18]) erfasst Achtsamkeit im Umgang mit sich selbst sowie das Maß der Akzeptanz und Präsenz auf einer Skala von 1,00 („nie“) bis 4,00 („oft“).

Zusätzlich wurden zu t0 soziodemografische Daten sowie Angaben zur derzeitigen Arbeitsbelastung und zur Vorerfahrungen mit anderen Entspannungsverfahren erhoben. Zudem wurden zu t1 einige Fragen zur Zufriedenheit mit dem Programm gestellt: Die TeilnehmerInnen konnten dabei ihren persönlichen Nutzen der Weiterbildungsreihe reflektieren, indem sie ihre Einschätzung zu Fragen wie z. B. „Die Teilnahme hat mir geholfen, wieder mehr Freude an der Arbeit zu verspüren“ mitteilten.

### Stichprobe

Untersucht wurden 44 Lehrkräfte unterschiedlicher Schulformen aus ganz Deutschland. Aufgrund unvollständiger Angaben von 8 TeilnehmerInnen beziehen sich die Ergebnisse auf 36 Lehrkräfte, von denen 15 über den AKiJu (Berlin) und 21 über das AVE (Frankfurt) teilnahmen. Sie waren im Alter von 28–59 (42,35 ± 8,21) Jahren und überwiegend weiblich (86 %). Die durchschnittliche Arbeitszeit wurde mit 37,26 ± 13,53 Wochenstunden angegeben. Die soziodemographischen Angaben sind in Tab. 1 beschrieben.

**Tab. 1:** Basisdaten aller untersuchten TeilnehmerInnen zum Messzeitpunkt t0, sowie im Gruppen- und Geschlechtervergleich

Messgröße		Gesamt (*n* = 36)		AKiJu (*n* = 15)		AVE (*n* = 21)		Männlich (*n* = 5)		Weiblich (*n* = 31)	
		*n*	M ± SD bzw. %	*n*	M ± SD bzw. %	*n*	M ± SD bzw. %	*n*	M ± SD bzw. %	*n*	M ± SD bzw. %
Altersdurchschnitt		31	42,35 ± 8,21	13	44,46 ± 8,71	18	40,83 ± 7,72	5	41,40 ± 7,40	26	42,54 ± 8,48
Geschlecht	Männlich	36	5 (14 %)	15	2 (13 %)	21	3 (14 %)	–	–	–	–
	Weiblich		31 (86 %)		13 (87 %)		18 (86 %)		–		–
Institution	AKiJu	–	–	–	–	–	–	5	2 (40 %)	31	13 (42 %)
	AVE		–		–		–		3 (60 %)		18 (58 %)
Durchschnittliche Arbeitswochenstunden		35	37,26 ± 13,53	15	37,33 ± 12,56	20	37,20 ± 14,54	5	40,20 ± 12,70	30	36,77 ± 13,81
Wie beurteilen Sie Ihre Arbeitsbelastung?	Gering	34	0 (0 %)	15	0 (0 %)	19	0 (0 %)	5	0 (0 %)	29	0 (0 %)
	Mäßig		8 (24 %)		4 (27 %)		4 (21 %)		1 (20 %)		7 (24 %)
	Eher hoch		18 (53 %)		7 (47 %)		11 (58 %)		2 (40 %)		16 (55 %)
	Sehr hoch		8 (24 %)		4 (27 %)		4 (21 %)		2 (40 %)		6 (21 %)
Fühlen Sie sich von Ihrer Arbeit überlastet?	Fast nie	35	0 (0 %)	14	0 (0 %)	21	0 (0 %)	4	0 (0 %)	31	0 (0 %)
	Selten		17 (49 %)		7 (50 %)		10 (48 %)		2 (50 %)		15 (48 %)
	Meistens		17 (49 %)		6 (43 %)		11 (52 %)		2 (50 %)		15 (48 %)
	Immer		1 (3 %)		1 (7 %)		0 (0 %)		0 (0 %)		1 (3 %)
Verfügen Sie, Ihrer Meinung nach, über angemessene Strategien zur Stressbewältigung?	Nein	36	1 (3 %)	15	0 (0 %)	21	1 (5 %)	5	0 (0 %)	31	1 (3 %)
	Eher nein		14 (39 %)		3 (20 %)		11 (52 %)		2 (40 %)		12 (39 %)
	Eher ja		16 (44 %)		9 (60 %)		7 (33 %)		2 (40 %)		14 (45 %)
	Ja		5 (14 %)		3 (20 %)		2 (10 %)		1 (20 %)		4 (13 %)
Ich habe bereits ähnliche (achtsamkeitsbasierte) Entspannungsverfahren praktiziert	Nein	36	8 (22 %)	15	2 (13 %)	21	6 (29 %)	5	0 (0 %)	31	8 (26 %)
	Ja		28 (78 %)		13 (87 %)		15 (71 %)		5 (100 %)		23 (74 %)
Ich führe regelmäßig Achtsamkeitsübungen durch	Nein	36	4 (11 %)	15	0 (0 %)	21	4 (19 %)	5	0 (0 %)	31	4 (13 %)
	(Fast) nie		3 (8 %)		0 (0 %)		3 (14 %)		1 (20 %)		2 (7 %)
	Gelegentlich		7 (19 %)		3 (20 %)		4 (19 %)		3 (60 %)		4 (13 %)
	Oft		7 (19 %)		3 (20 %)		4 (19 %)		0 (0 %)		7 (23 %)
	Häufig		4 (11 %)		2 (13 %)		2 (10 %)		0 (0 %)		4 (13 %)
	(Fast) täglich		11 (31 %)		7 (47 %)		4 (19 %)		1 (20 %)		10 (32 %)

*AKiJu* Achtsamkeit für Kinder und Jugendliche, *AVE* Institut für Achtsamkeit, Verbundenheit und Engagement, *M* Mittel, *SD* Standardabweichung

### Analysen

Die statistische Auswertung erfolgte mit IBM® SPSS® Statistics (Version 26; IBM SPSS Statistics V.23.0, IBM, Armonk, NY, USA). Weiterbildungsgruppen- bzw. Geschlechtsunterschiede der Parameter zu t0 wurden mittels ungepaartem t‑Test (bzw. Mann-Whitney-U-Test mit exakter Signifikanz bei einzelnen nicht-gleichverteilten Messgrößen und *n* < 30) überprüft. Zur Analyse der Prä-Post-Veränderung der Outcomevariablen wurden Mittelwertvergleiche von t0 und t1 mittels gepaartem t‑Test (bzw. Wilcoxon-Test mit exakter Signifikanz) durchgeführt. Die Höhe der Veränderung von t0 zu t1 (Δ_t1–t0_) wurde wiederum über ungepaarte t‑Tests (bzw. Mann-Whitney-U) auf Gruppen- und Geschlechtsunterschiede untersucht. Zur Interpretation dienten die üblichen Maße: Signifikanzwert *p*, t‑Wert und Freiheitsgrade df (letztere können nur im Fall von parametrischen Tests angegeben werden). Zur Beurteilung der Effektstärke wurde zudem Cohen’s d und d_z_ (= $\frac{2t}{\sqrt{df}}$) für parametrische bzw. der Korrelationskoeffizient r nach Pearson für nichtparametrische Tests berechnet.

Um zu prüfen, ob die Vorerfahrung mit Entspannungsverfahren Einfluss auf die Wirksamkeit der Weiterbildung (Δ_t1-t0_) hat, wurden ungepaarte t‑Test (bzw. Mann-Whitney-U) gerechnet.

Die abschließende Beurteilung der Weiterbildung durch die TeilnehmerInnen zu t1 wurde deskriptiv ausgewertet. Die Angaben „stimme voll und ganz zu“ und „stimme zu“ wurden hierbei angesichts des für die Praxis geringen interpretativen Unterschiedes zusammengefasst.

## Ergebnisse

### Baseline und initiale Gruppenunterschiede

Die teilnehmenden Lehrkräfte schätzten ihre Belastung und ihr Arbeitspensum zu Beginn hoch ein. 77 % der Lehrkräfte gaben eine hohe Arbeitsbelastung an, über die Hälfte fühlte sich von ihrer Arbeit ständig überlastet. 42 % verfügten laut eigener Einschätzung nicht über eine geeignete Methode zur Stressbewältigung. 78 % der TeilnehmerInnen gaben an, über Vorerfahrungen mit achtsamkeitsbasierten Entspannungsverfahren zu verfügen, praktizierten aber in sehr unterschiedlicher Häufigkeit (Tab. 1). Der TM zeigte initial eine kritische Stressbelastung von 3,50 ± 0,74. Der PSQ ergab im Vergleich mit der Referenzstichprobe [6] einen hohen Stressgesamtwert von 2,48 ± 0,44 sowie auffallend hohe Maße an Anspannung (2,69 ± 0,55) und Anforderungen (2,73 ± 0,53). Das allgemeine Wohlbefinden bzw. die Lebensqualität der Lehrkräfte war dementsprechend vergleichsweise niedrig (13,76 ± 2,82; Tab. 2).

**Tab. 2:** Prä-Post-Veränderungen der psychischen Gesundheitswerte der Gesamtstichprobe (gepaarter t‑Test)

Messgröße (*n* = 31)	M		SD		*p*	t	Df	D_z_	Δ_t1–t0_	
	t0	t1	t0	t1					M	SD
PSQ_Gesamt	2,48	2,15	0,44	0,37	< 0,001*	4,91	30	0,88	−0,33	0,38
PSQ_Sorgen	2,15	1,79	0,50	0,37	< 0,001*	5,38	30	0,97	−0,36	0,37
PSQ_Anspannung	2,69	2,20	0,55	0,42	< 0,001*	5,29	30	0,95	−0,47	0,49
PSQ_Freude	2,61	2,90	0,53	0,42	0,001*	−3,72	30	−0,67	0,30	0,44
PSQ_Anforderungen	2,73	2,52	0,53	0,52	0,035*	2,21	30	0,40	−0,21	0,52
„Tedium measure“	3,50	3,11	0,74	0,66	0,001*	3,63	30	0,65	−0,39	0,60
WHO‑5	13,76	15,83	2,82	3,00	< 0,001*	−4,56	30	−0,82	2,07	2,53
MAAS	2,21	2,48	0,45	0,37	0,002*	−3,40	30	−0,61	0,28	0,45
FAA_Gesamt	3,01	3,40	0,41	0,39	< 0,001*	−5,73	30	−1,03	0,39	0,38
FAA_Präsenz	3,14	3,51	0,45	0,43	< 0,001*	−5,63	30	−1,01	0,37	0,37
FAA_Akzeptanz	2,90	3,31	0,45	0,43	< 0,001*	−4,98	30	−0,89	0,41	0,46

*Signifikante Werte markiert

*PSQ* Perceived Stress Questionnaire, *TM* Tedium measure, *WHO‑5* Wohlbefindensindex, *MAAS* Mindful Attention and Awareness Scale, *FFA* Freiburger Fragebogen zur Achtsamkeit

Zum Zeitpunkt t0 konnten Unterschiede zwischen den beiden Gruppen in Berlin und Frankfurt festgestellt werden: Bei vergleichbarer Arbeitszeit gaben die Gruppen ein ähnliches Ausmaß an Be- bzw. Überlastung an. Die meisten Teilnehmenden in Berlin (80 %) verfügten bereits über Vorerfahrungen mit Stressbewältigungsmethoden, die Frankfurter Lehrerschaft hingegen überwiegend nicht (57 %). Außerdem praktizierten sie im Vergleich bereits häufiger Achtsamkeitsübungen (Tab. 1). Auch zeigten die Teilnehmenden in Berlin durchgängig bessere psychische Gesundheitswerte mit signifikanten Unterschieden für die Parameter Sorgen (*p* = 0,033), Anspannung (t[34] = −2,08, *p* = 0,046), Achtsamkeit (FFA_gesamt_: t[34] = 2,61, *p* = 0,013), Präsenz (t[34] = 2,21, *p* = 0,034) und Akzeptanz (*p* = 0,11) im Vergleich zu den TeilnehmerInnen aus Frankfurt.

Im Geschlechtsvergleich gaben die Lehrer etwa 3,5 Wochenarbeitsstunden mehr (40,20 ± 12,70) und weniger persönliche Achtsamkeitspraxis an als die Lehrerinnen (Tab. 1), es zeigten sich aber keine statistisch signifikanten Unterschiede in den abgefragten Parametern zur psychischen Gesundheit.

### Veränderungen von t0 zu t1

Aus dem Prä-Post-Vergleich (Tab. 2) der gesamten Stichprobe ergaben sich signifikante Veränderungen aller Messgrößen mit überwiegend mittleren und großen Effektstärken. Große Effekte zeigten sich v. a. hinsichtlich Gesamtstressempfinden (t[30] = 4,91, *p* < 0,001, d_z_ = 0,88), Sorgen (t[30] = 5,38, *p* < 0,001, d_z_ = 0,97) und Anspannung (t[30] = 5,29, *p* < 0,001, d_z_ = 0,95), Wohlbefinden (t[30] = −4,56, *p* < 0,001, d_z_ = −0,82) sowie Achtsamkeit (FFA_gesamt_: t[30] = −5,73, *p* < 0,001, d_z_ = −1,03), Präsenz (t[30] = −5,63, *p* < 0,001, d_z_ = −1,01) und Akzeptanz (t[30] = −4,98, *p* < 0,001, d_z_ = −0,89).

Das Stressempfinden der Lehrkräfte verringerte sich um durchschnittlich 0,33 ± 0,38 Punkte (8 %), Sorgen (Δ_t1–t0_ = −0,36 ± 0,38) und Anspannung (Δ_t1–t0_ = −0,47 ± 0,37) nahmen um 9 % bzw. 12 % ab. Die Gefahr, ein Burn-out zu entwickeln, sank (TM: Δ_t1–t0_ = −0,39 ± 0,60). Ihr Wohlbefinden (13,76 ± 2,82) stieg um 8 % auf 15,83 ± 3,00. Die Achtsamkeit (FFA_gesamt_: Δ_t1–t0_ = 0,39 ± 0,38), Präsenz (Δ_t1–t0_ = 0,37 ± 0,37) und Akzeptanz (Δ_t1–t0_ = 0,41 ± 0,46) der TeilnehmerInnen nahm um jeweils etwa 10 % zu.

Trotz unterschiedlicher Ausgangswerte konnten hinsichtlich der Höhe der Prä-Post-Veränderungen keine statistisch signifikanten Unterschiede im Vergleich zwischen den Berliner und Frankfurter TeilnehmerInnen festgestellt werden. Die Lehrerinnen und Lehrer unterschieden sich ebenfalls nicht-signifikant in den Ausprägungen der Veränderungen von t0 zu t1.

Die t‑Tests zum Vergleich der Lehrkräfte mit und ohne Vorerfahrung in Entspannungsverfahren ergaben keine Unterschiede von Δ_t1–t0_ der Parameter_._

### Bewertung der Weiterbildung

Am Ende des Programms zu t1 wurde auch die AISCHU-Weiterbildung rückblickend evaluiert. Alle TeilnehmerInnen empfanden diese für sich persönlich (100 %) wie auch für ihre Schule (96 %) als „sehr wertvoll“. Konkret verhalf die Teilnahme den meisten Lehrkräften dabei, die Fähigkeit zur Entspannung (93 %) und Stressbewältigung (86 %) zu fördern. Außerdem beeinflusste sie das kollegiale Miteinander und die Beziehung zur Klasse (86 %) sowie die Stimmung am Arbeitsplatz (60 %) positiv. Daneben gaben die Teilnehmenden an, fokussierter (75 %) und mit mehr Freude (74 %) zu arbeiten und eine positive Einstellung zu Herausforderungen entwickelt zu haben (96 %).

## Diskussion

In dieser explorativen Pilotstudie der AISCHU-Weiterbildungskomponente für LehrerInnen wurde basierend auf Selbstaussagen der teilnehmenden Lehrerkräfte eine deutliche Verbesserung im Hinblick auf Stressbewältigung, Belastung, Achtsamkeit und Burn-out-Gefährdung festgestellt. Vor dem Hintergrund der initial berichteten hohen Stress- und Belastungswerte ist dies ein vielversprechender Befund, wenngleich die Wirksamkeit aufgrund einer fehlenden Kontrollgruppe nicht vergleichend beurteilt werden kann. In Pilotstudien, die in über keine Kontrollgruppe verfügen, werden die Effektstärken häufig überschätzt. Dennoch ist der Vergleich mit Studien mit Kontrollgruppe sinnvoll, weil dadurch eine realistische Einordnung der Wirksamkeit ermöglicht wird. Insgesamt zeigen sich in unserer Studie erwartungsgemäß die größten Effekte bei der Verbesserung von Achtsamkeit. Der Einfluss auf psychisches Wohlbefinden und Belastung liegen ebenfalls im hohen bis mittleren Effektstärkebereich. Die Verringerung von Sorgen und Anspannung fällt dabei am höchsten aus, während Steigerung der Freude sowie die Reduktion von Anforderungen erwartungsgemäß geringer ausfällt. Unsere Untersuchung zeigte geschlechtsunabhängig mittlere bis große Effekte für die analysierten Messgrößen. Die in unserer Pilotstudie festgestellten Effektstärken lassen im Vergleich mit anderen Studien [4, 11] durchaus den Rückschluss zu, dass das AISCHU-Programm sowohl in einem offenen, selbstgewählten, als auch in einem geschlossenen, über das Schulamt angebotenen Format hohe Wirksamkeit erzielen kann. Dies ist insofern bemerkenswert, weil trotz Anzeichen für Selbstselektionseffekte, wie beispielsweise höher belastete und achtsamkeitsaffinere TeilnehmerInnen in der Selbstzahlergruppe, eine vergleichbare Veränderung der beobachteten Parameter in beiden Gruppen festgestellt wurde. Klingbeil und Renshaw [11] stellten in ihrer Metaanalyse einen mittleren Gesamtinterventionseffekt von Achtsamkeitsinterventionen für Lehrkräfte heraus, was für sowohl für die Wirksamkeit als auch Praxistauglichkeit achtsamkeitsbasierter Programme spricht.

Sicherlich war die Stichprobe der teilnehmenden Lehrenden nicht repräsentativ, da von ihnen 78 % Vorerfahrungen im Zusammenhang mit achtsamkeitsbasierten Entspannungsverfahren aufwiesen. Interessanterweise unterschieden sich die Wirkeffekte (Δ_t1–t0_) der TeilnehmerInnen mit und ohne Vorerfahrung nicht voneinander. Dabei fällt jedoch auf, dass nur 58 % der Teilnehmenden angeben haben, über eine geeignete Methode der Stressbewältigung zu verfügen. Das deutet darauf hin, dass die Vorerfahrung einiger Teilnehmenden eher theoretischer Natur war. Für diese Interpretation spricht auch, dass nur 42 % der Teilnehmenden angegeben haben, häufig oder täglich Achtsamkeit zu praktizieren. Allerdings legen weitere Korrelationsanalysen nahe, dass besonders Personen mit fehlenden bzw. suboptimalen Stressbewältigungsstrategien einen größeren Nutzen aus der Weiterbildung ziehen könnten. Durch die programmatische Verankerung innerhalb der LehrerInnenausbildung und Integration in das schulische Gesundheitsmanagement könnten speziell auch jene Lehrkräfte erreicht werden, die bislang keinen Zugang zu achtsamkeitsbasierten Angeboten haben, um präventive Effekte auch im Sinne der Burn-out-Prophylaxe zu erzielen.

Vor dem Hintergrund der aktuellen COVID-19-Krise („coronavirus disease 2019“) könnten achtsamkeitsbasierte Programme wie AISCHU Lehrkräfte im Umgang mit erhöhter psychischer Belastung unterstützen. Aufgrund der Belastungen durch Home-Schooling, aber auch durch die Sorge selbst zu erkranken hat das hohe Ausmaß von Stress während der Coronakrise bei Lehrkräften noch einmal zugenommen [6]. In diesem Zusammenhang ist es wichtig festzuhalten, dass die hohen Belastungswerte der TeilnehmerInnen unserer Studie vor der Coronakrise eroben wurden. Aufgrund der hohen initialen Belastungswerte, aber auch Erfahrungen im klinischen bzw. psychotherapeutischen Kontext ist davon auszugehen, dass das Programm auch im Sinne der sekundären Prävention wirksam ist. Aufgrund des Infektionsschutzes in Pandemiezeiten ist es folgerichtig auch über den Einsatz digitaler oder hybrider Formate nachzudenken. Eine aktuelle Studie aus Italien hat die Wirksamkeit eines achtsamkeitsbasierten Onlineprogramms für Lehrkräfte untersucht [14]. Innerhalb von 8 Wochen konnten mit je 2 Onlinekursstunden pro Woche und 30 min selbständiger Praxis positive Effekte auf das psychische Wohlbefinden, das Burn-out-Risiko sowie Angst und Unsicherheit von Lehrkräften – v. a. mit geringerer Selbstregulationsfähigkeit und Resilienz – festgestellt werden.

Jedoch sollte der Mehrwert von achtsamkeitsbasierten Programmen im Schulkontext sowohl für SchülerInnen als auch LehrerInnen auch krisenunabhängig v. a. als primärpräventive und gesundheitsförderliche Maßnahme für SchülerInnen [3, 19] und Lehrkräfte [4, 13, 14] betont werden. Konkret läßt sich dadurch die Selbstregulationsfähigkeit erhöhen und als Konsequenz nicht nur das Risiko für Burn-out, Angst, Stress und Depressionen verringern, sondern auch Wohlbefinden Selbstwirksamkeit und Resilienz fördern. Unsere Befunde können bisherige Erkenntnisse zusätzlich stützen. Insofern plädieren wir für den systematischen Ausbau von achtsamkeitsbasierten Programmen im Kontext Schule zum Wohle von SchülerInnen und Lehrpersonal sowie die entsprechende Schulung der Lehrkräfte idealerweise möglichst früh in ihrer Ausbildung.

## Limitationen

Hinsichtlich der allgemeinen Aussagekraft unserer Ergebnisse müssen einige Aspekte unserer explorativen Pilotstudie limitierend berücksichtigt werden. Durch die vergleichsweise kleine Stichprobe und den fehlenden Vergleich mit einer Kontrollgruppe ist die Wirksamkeit der Intervention nicht abschließend zu bestimmen. Da die Anmeldung an persönliches Interesse geknüpft war und die meisten TeilnehmerInnen bereits Erfahrung in Achtsamkeitspraxis hatten, handelte es sich vermutlich um besonders empfängliche Probanden und somit selbstselektierte achtsamkeitsaffine Lehrkräfte. Da keine Angaben zum im AISCHU-Konzept vorgesehenen Erfahrungsaustausch der TeilnehmerInnen zwischen den Seminarterminen erhoben wurden, kann der Nutzen bzw. Einfluss dieses Aspekts von kollegialem Austausch nicht beurteilt werden. Außerdem kann nicht ausgeschlossen werden, dass weitere Faktoren innerhalb des Studienzeitraums die Untersuchungsergebnisse beeinflusst haben. Darüber hinaus wurde in dieser Studie auch nicht untersucht, wie langfristig die erzielten Effekte auch nach Abschluss der Weiterbildung erhalten bleiben. Diese Punkte könnten und sollten aber aufgrund der vielversprechenden Ergebnisse in einer umfassenderen Studie untersucht werden.

## Fazit für die Praxis

- In dieser explorativen Pilotstudie zu AISCHU (Achtsamkeit in der Schule) für Lehrkräfte konnten erstmals vielversprechende Hinweise auf die Wirksamkeit im Sinne von Stressreduzierung, Burn-out-Risikominimierung sowie Verbesserung der Lebensqualität bei relativ hoch belasteten Lehrkräften beobachtet werden.
- Obwohl noch weiterer Forschungsbedarf besteht, kann zum gegenwärtigen Zeitpunkt ein unmittelbarer Nutzen für die Teilnehmenden abgeleitet werden. Daher sollten achtsamkeitsbasierte Interventionen wie das AISCHU-Programm speziell für Lehrkräfte zukünftig vermehrt in der Praxis eingesetzt werden und Aus‑, Fort- und Weiterbildungsprogramme dementsprechend angepasst werden.

## Supplementary Information

AISCHU-Programm für LehrerInnen
