# Supplementary material for: Mindfulness in school—evaluation of the special stress reduction program AISCHU for teachers
Source: Pravent Gesundh. 2021 Jul 5;17(3):299–305. [Article in German] doi: 10.1007/s11553-021-00870-9 (PMC8256641; doi:10.1007/s11553-021-00870-9)
Supplement: Supplementary file 1 — AISCHU-Programm für LehrerInnen [file 11553_2021_870_MOESM1_ESM.docx]

Tabelle 1: **Inhalt und Ablauf des Programms**

| - **Für alle Seminarteile gilt, dass im Seminarablauf eine enge Verbindung zwischen theoretischem Input und Übungspraxis umgesetzt wird.** - **Fester Bestandteil in jeder Session: Achtsamkeitsübungen im Sitzen, Achtsames Bewegen (Qigong), Geführte Imagination zum jeweiligen Thema zur Selbstbeobachtung** - **Die angebotenen Übungsformen werden immer auch im Hinblick auf Anwendungen im Unterricht reflektiert** - **Die Übungen sind verfügbar in: Kartenset Achtsamkeit, Weinheim: Beltz 2020**   **Zwischen den Seminarterminen wird in Dyaden mit Selbstbeobachtungsaufgaben gearbeitet** | |
| --- | --- |
| Die Haltung der Achtsamkeit  10 Stunden | **Theorieteil:**  Schwerpunkte der Achtsamkeitsforschung  Wirkungsmechanismen von körper- und erfahrungsorientierten Interventionen  Grundlegende Informationen über den Aufbau des Gehirns, Mind-Body-Prozesse, Propriozeption, Embodiment  Erfahrungsorientierung – Perspektive der Ersten Person  **Praxisteil**  Unterschiedliche Übungen zur Atembeobachtung  (Trainieren der Rückholvorgangs, Aufmerksamkeitssteuerung) |
|  |  |
| Stressbewältigung  10 Stunden | **Theorieteil:**  Unterschiedliche Stress-Theorien, Fokus auf dem Ansatz von Lazarus  Negativity Bias  Umgang mit Wut und Angst  Stressphysiologie, Möglichkeiten der Selbststeuerung,  Bedeutung des Atems – Vegetatives Nervensystem  **Praxisteil**  Selbsterfahrung – Erkunden der persönlichen Stressoren  Persönliche Auslöser für Fight/Flight/Freeze wahrnehmen  Kognitive Übungen zur Selbstregulation/ zum Gegensteuern bei Rumination  Die Methode RAIN von Jack Kornfield |
| Die Tücken des Belohnungssystems  10 Stunden | **Theorieteil**  Habit-Loops neurophysiologisch erläutern  Substanzgebundene Süchte und stoffungebundene Süchte  erläutern  Interventionsmöglichkeiten erläutern  **Praxisteil**  Gewohnheitsmuster erkunden, die persönlichen Spannungslöser erkennen und mit dem Craving umgehen lernen  Strategien für den Umgang mit digitalen Medien im Alltag  Übungen zum „Achtsamen Essen“ und generell zu einer bewussten, gesunden Lebensführung |

| Ressourcenorientierung  10 Stunden | **Theorieteil**  Grundlegende Aspekte der Positiven Psychologie  „Die Macht der guten Gefühle“  Freude als Ressource  Self-Compassion – Umgang mit negativem Self-Talk  Methoden der Selbstberuhigung/ Reframing etc.  **Praxisteil**  Übungen zur Wahrnehmung der persönlichen Ressourcen  „Die Macht der guten Gefühle“  Die Methode HEAL von Rick Hanson  Achtsames Schreiben  Wahrnehmungsschulung/ Kreativitätserkundungen |
| --- | --- |
| Beziehungsgestaltung  10 Stunden | **Theorieteil**  Informationen aus der Bindungsforschung  Bedeutung von Projektionen und Vorurteilsstrukturen  Resonanzphänomene/ Spiegelneuronenforschung  Forschung zum Training von Mitgefühl (Compassion)  **Praxisteil**  Der Achtsame Dialog  Spiegelübungen  Das Achtsame Quartett  Reflektion der Dyaden-Arbeit der vorgegangenen Zeit |
| Achtsamer Alltag  10 Stunden | **Theorieteil**  Überblick über das Salutogenese-Konzept  Elemente der Gesunderhaltung im Alltag  Zusammenfassung der Aspekte der Weiterbildung im Sinne der nachhaltigen Integration in den Alltag  **Praxisteil**  Übungen zur Phrasierung des Alltags (Work-Life-Balance)  Achtsames Essen  Kreativitätsübungen, Visualisierungen  Achtsames Schreiben, Anregungen zum regelmäßigen Journalling |
